# Tissue Sampling and Homogenization in the Sub-Microliter Scale with a Nanosecond Infrared Laser (NIRL) for Mass Spectrometric Proteomics

**DOI:** 10.3390/ijms221910833

**Published:** 2021-10-07

**Authors:** Jan Hahn, Manuela Moritz, Hannah Voß, Penelope Pelczar, Samuel Huber, Hartmut Schlüter

**Affiliations:** 1 Section/Core Facility Mass Spectrometry and Proteomics, University Medical Center Hamburg-Eppendorf (UKE), Martinistr. 52, 20246 Hamburg, Germany; ma.moritz@uke.de (M.M.); ha.voss@uke.de (H.V.); h.schluet@uke.de (H.S.); 2 Section of Molecular Immunology and Gastroenterology, I. Department of Medicine, University Medical Center Hamburg-Eppendorf (UKE), Martinistr. 52, 20246 Hamburg, Germany; p.pelczar@uke.de (P.P.); s.huber@uke.de (S.H.)

**Keywords:** tissue sampling, tissue homogenization, nanosecond infrared laser, laser ablation, proteomics, mass spectrometry

## Abstract

It was recently shown that ultrashort pulse infrared (IR) lasers, operating at the wavelength of the OH vibration stretching band of water, are highly efficient for sampling and homogenizing biological tissue. In this study we utilized a tunable nanosecond infrared laser (NIRL) for tissue sampling and homogenization with subsequent liquid chromatography tandem mass spectrometry (LC-MS/MS) analysis for mass spectrometric proteomics. For the first time, laser sampling was performed with murine spleen and colon tissue. An ablation volume of 1.1 × 1.1 × 0.4 mm³ (approximately 0.5 µL) was determined with optical coherence tomography (OCT). The results of bottom-up proteomics revealed proteins with significant abundance differences for both tissue types, which are in accordance with the corresponding data of the Human Protein Atlas. The results demonstrate that tissue sampling and homogenization of small tissue volumes less than 1 µL for subsequent mass spectrometric proteomics is feasible with a NIRL.

## 1. Introduction

In mass spectrometry-based proteomic analysis of tissues, sampling and homogenization is one of the most challenging steps, aiming at a complete release and solubilization of all proteins present in the cells and their compartments within the intact tissue before its sampling and homogenization [1]. In particular, structural interactions of proteins and the formation of macromolecular assemblies make it challenging to fully solubilize proteins from tissue [2]. When choosing a method for homogenization, the high degree of heterogeneity of the chemical properties of proteins should also be considered. This is particularly important in the analysis of tissues, where there are many different types of cells performing specific functions in the tissue [2,3]. Tissue homogenization can be divided into two steps: tissue disruption and cell lysis [2]. Common methods for tissue disruption are mechanical homogenization, including vortexing and grinding with different beads, sonication, pressure-cycling technology or liquid nitrogen treatment and subsequent grinding of the frozen tissue. In the second step of homogenization, detergents or physical methods, such as osmotic shock, mechanical blending, sonication, and/or freeze/thaw treatment can be used for cell lysis [2]. Depending on the particular aim of the analysis, a combination of detergents and mechanical methods is required for the homogenization of tissues. However, when choosing detergents, it is essential to ensure that they are suitable for chromatography and mass spectrometry (MS) and have no interfering or suppressing effect [2].

Furthermore, the duration of the applied homogenization method is also critical and often a very time-consuming process. To release the proteins from the intracellular compartments, tissue and cell lysis steps are applied during homogenization, which release proteases and other enzymes. These biological catalysts can lead to changes in post-translational modifications (PTMs) or even complete degradation of the proteins over time [3]. The PTMs of proteins play a significant role in many diseases. Therefore, if a tissue proteome is to be studied, it is even more important to be able to characterize the proteoforms as they are present in their native tissue environment. While there are many methods, such as using protease inhibitors, to prevent proteome changes due to proteolytic processes, these do not address all enzymes, resulting in at least partial conversion of proteoforms [4].

Pressure BioSciences Inc. has developed a pressure-cycling technology (PCT) for the extraction of proteins from cells and tissues. Cell lysis is caused by rapid alternating cycles of high and low pressures. In comparison to conventional homogenization techniques like the probe sonicator and bead mill, the reaction chambers are temperature-controlled, resulting in no excessive heat during homogenization, which could possibly lead to changes in PTMs [5].

Laser capture microdissection (LCM) represents another method for tissue sampling [6,7]. With this method, cells from a specific area in a tissue can be selected using a microscope and isolated from the tissue by a near-infrared laser energy pulse, transferring it to an adhesive polymer film [3,8]. Next, the polymer is removed from the tissue with the bound cells of interest attached. With appropriate extraction buffers, the cells are released from the polymer surface and the proteins can be subjected to proteolytic digestion for bottom-up proteomics [8]. The advantage of this method is the possibility of selecting cell areas of interest after examination of the tissue section via the microscope, which is particularly useful in clinical applications, for example to distinguish tumor tissue from benign tissue [3]. However, this method is quite time-consuming and can only be applied to two-dimensional tissue sections. Stacking of sequential sections for a three-dimensional view is possible, but very challenging and expensive.

A novel approach for tissue sampling emerged from irradiation with an infrared laser (IR) emitting light at a wavelength of 2.94 µm. The energy of the IR laser is absorbed by water molecules and immediately converted into translational energy caused by the vibrational motion of their OH stretch band, resulting in an explosion of the irradiated water molecules, which leads to the decomposition of the tissue and thereby to the transfer into the gas phase [9]. In the case of the picosecond infrared laser (PIRL) the transfer of the energy of PIRL into translational energy is much faster than the transfer into thermal energy [10,11,12]. Because of this process, tissue irradiated with PIRL is transferred into an aerosol by cold vaporization [13].

The tissue aerosol represents an ideal homogenate, which can be used for subsequent analytical approaches such as mass spectrometric proteomics. Compared to mechanical homogenization, this is a very gentle method of sample extraction and homogenization, avoiding time-consuming preparation steps. Over the past decade, this tissue sampling and homogenization has been successfully demonstrated with a PIRL, nanosecond infrared laser (NIRL) and even a high-energy microsecond infrared laser (MIRL) with subsequent mass spectrometric proteomics [4,14,15,16,17,18,19,20,21] or directly coupled to real-time MS instruments, such as the “SpiderMass” technology [22,23,24,25,26].

In our previous studies we utilized a picosecond infrared laser (PIRL) [4,13,16,19] as well as a microsecond infrared laser (MIRL) [15,16] for tissue sampling and homogenization for subsequent proteomics and lipidomics. In a first study by Kwiatkowski et al. (2015), it was shown that tissue sampling with the PIRL makes proteins accessible in a wide range from a few kilodaltons to several million daltons. Furthermore, it was demonstrated that post-translational modifications like glycans of glycoproteins were not lost by PIRL ablation. Kwiatkowski et al. also confirmed that proteins are not denatured during PIRL ablation, since enzymatic activity was detectable after irradiation of samples with PIRL [13].

Kwiatkowski et al. (2016) focused on the investigation of proteoforms in tissues. Using human tonsil and rat pancreas tissue, a comparison of mechanical homogenization (cryogrinder or bead mill with 3 mm stainless steel beads) with PIRL ablation showed that the latter contained much more intact proteoforms and a larger number of identified proteins than the mechanical homogenate. Thus, tissue sampling with the PIRL laser yields not only a higher number of identified proteins, but also access to the intact proteoforms as they exist in the intact tissue [4].

In the study of Hänel et al. (2018), the MIRL was demonstrated as a further method for tissue sampling for mass spectrometric proteomics. In that work, for the first time an IR laser was used for tissue sampling of xenograft primary tumors and paired spontaneous metastases. In contrast to PIRL, MIRL is based on much longer pulse durations during laser ablation, ranging within microseconds rather than picoseconds. Based on mass spectrometry proteomic analysis of the MIRL ablated ovarian and liver metastases, some new presumed drivers in metastasis formation were identified, which may be used as new targets for functional studies [15].

In a publication by Krutilin et al. (2019), the PIRL and MIRL were directly compared. Muscle, liver, and kidney tissues of rats were examined. Krutilin et al. showed that both laser systems are suitable for tissue sampling and homogenization. A larger yield of proteins, identified by bottom-up proteomics, was obtained with the PIRL for liver tissue and with the MIRL for muscle tissue. Regarding the enzymatic activity of the proteins based on ablated kidney tissue, it was shown that proteins are denatured during tissue sampling with the MIRL, whereas the PIRL ablation had no denaturing effect. Due to the six orders of magnitude longer pulse duration and a much higher pulse energy, MIRL ablation heats the tissue causing denaturation of proteins in the cells neighboring the zone of ablated cells [16].

In this study, we applied the NIRL for the first time for the sampling of colon and spleen tissue. Unlike previous work, our laser setup is based on a wavelength tuneable NIRL with a pulse duration of about 7 ns. Just like PIRL and MIRL, the water molecules in tissue are excited by using a laser wavelength at the absorption peak of water around 2.94 µm. Highly energetic excitation of the OH vibrational stretching band transfers the sampled tissue into the gas phase, forming a plume of homogenized aerosol. In our setup, we utilize glass cover slips, which are placed slightly above the tissue during sampling to collect the plume as a condensate. In contrast to our previous studies, this approach reduces material loss by avoiding tubing and therefore allows lower sampling amounts, which increases the spatial resolution of the sampling location in the tissue. Our new setup represents an improvement in terms of miniaturization of tissue sampling towards an ablation volume of approximately 0.5 µL, which is roughly the size of a steel pinhead.

## 2. Results

**Figure 1:**
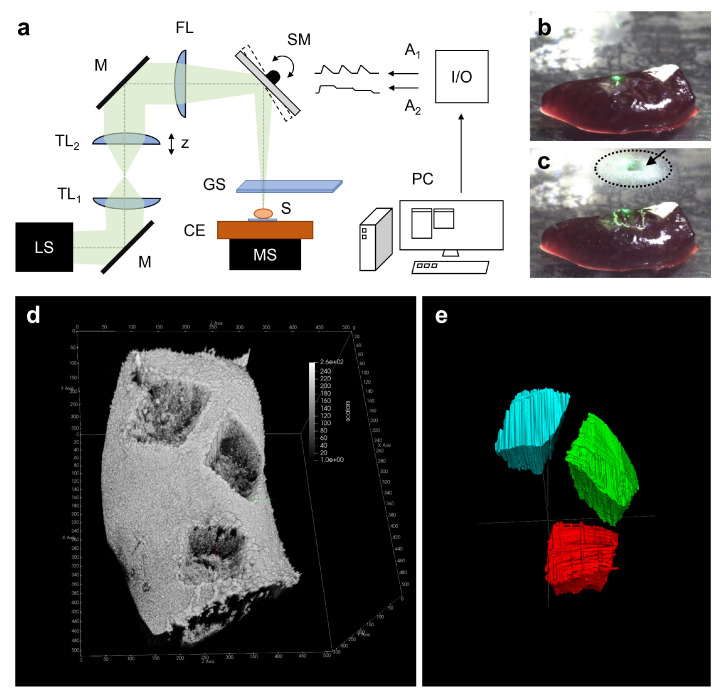
(**a**) Schematic of the ablation setup. Green: divergent beam; LS: NIRL laser system; M: silver mirror; TL_1_, TL_2_: telescope lenses; FL: focusing lens (of 150 mm focal length); SM: two-axis scanning mirror; S: scanning of the sample; MS: manual stage; CE: cooling element (−15 °C); I/O: input/output card; A_1_, A_2_: analog control signals; PC: computer. GS: glass cover slip; (**b**,**c**) Photos of the tissue before and after irradiation; (**c**) Dashed ellipse: Condensed tissue aerosol. Arrow: indicating an area of lost condensate. Green light: pilot laser (532 nm wavelength); (**d**) Rendered 3D OCT image of the reference ablations on formalin-fixed murine spleen for ablation volume measurement purposes with the open-source application ParaView 5.9.1 [27]; (**e**) Manually segmented ablation volumes (with the labels red, blue, green) in the open-source software ITK-Snap 3.8.0 [28].

A nanosecond infrared laser was used to sample fresh-frozen murine colon (*n* = 3) and spleen (*n* = 3) tissue with a beam scanning ablation setup, depicted in Figure 1a. The condensate of the tissue plume was collected by placing a glass cover slip directly over the sample during ablation, shown in Figure 1b,c. The divergent beam of a NIRL system is reflected via a silver mirror into a telescope, consisting of two focusing lenses, for collimation. A focusing lens of 150 mm focal length in combination with a two-axis scanning mirror was used to scan the sample on a manual stage, equipped with a cooling element (−15 °C). The scanning mirror is controlled by two analog signal lines of an input/output card connected to a computer. A glass cover slip on a manual three-axis stage is placed 2–4 mm above the ablation site to collect the aerosol. The tissue aerosol is condensed onto the glass cover slip. Within the area of the condensate, a square of the size of the ablation area, is missing after condensation. The condensate in that area was removed by the laser beam.

**Figure 2:**
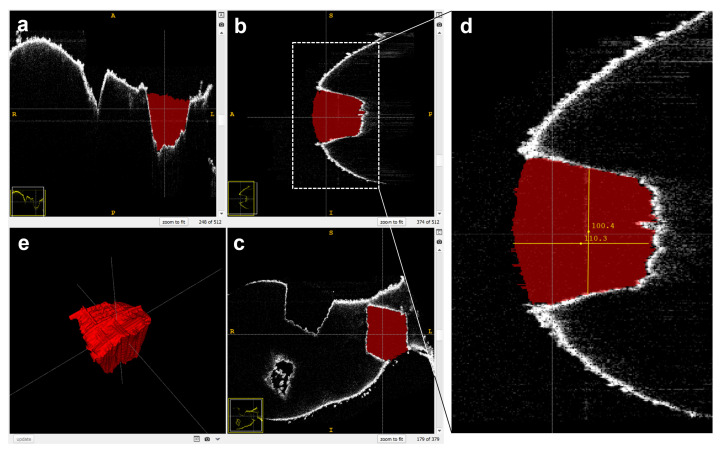
Example of 3D segmentation of an ablation site with the open-source software ITK Snap 3.8.0 [28]. (**a**–**c**) Slice views of the three dimensions; (**d**) Determination of the mean volume dimensions in pixels with the integrated measuring tool; (**e**) 3D rendering of the segmented volume.

Based on the 3D imaging by optical coherence tomography (OCT), image processing and segmentation, shown in Figure 1d,e, a mean ablation volume of 0.43 ± 0.06 µL was determined for tissue sampling; this example was performed on formalin-fixed spleen tissue (*n* = 3) for better handling. Furthermore, mean ablation dimensions for central width and depth were determined with the provided tools of the used segmentation software (Figure 2) to measure about 1.1 mm and 0.4 mm, respectively. In Table 1 we show the separate numbers of the ablation volumes and dimensions for the three ablation sites.

**Figure 3:**
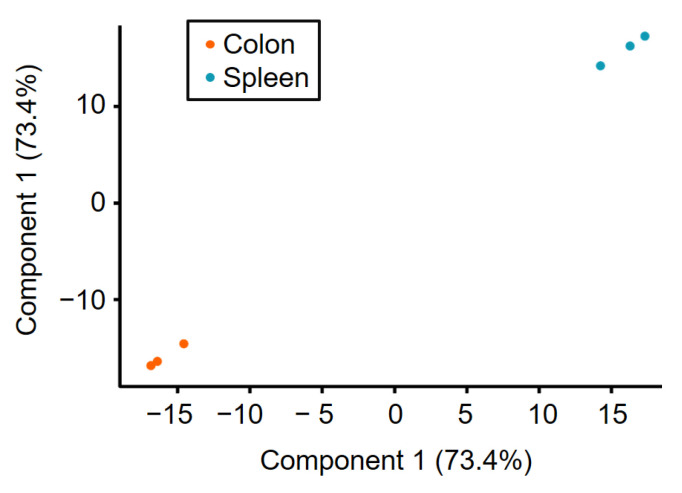
Scatter plot visualization of three murine colon (orange) and spleen (blue) tissue samples, showing component 1 against component 1 of PCA. The visualized data is based on 501 proteins with quantitative information for all samples (see Supplementary Table S3).

Differential quantitative proteome analysis of the murine colon tissue and the spleen tissue (three biological replicates from each tissue) sampled with NIRL resulted in 1889 identified proteins (see Supplementary Tables S1 and S2), where in a total of 501 proteins had quantitative data in all samples. In Figure 3, the results of a principal component analysis (PCA) (see Supplementary Table S3) are shown as scatter plot visualization, where component 1 (73.4%) is plotted against component 1 (73.4%). The PCA of the 501 proteins revealed a clear tissue-related distinction between colon and spleen samples based on component 1. Thus, the biological differences between the samples based on the different tissue types have the greatest influence. The biological replicates within a sample group play a subordinate role, as they show a low Euclidean distance in the scatter plot visualization of PCA, representing a high grade of similarity. This is also shown by the further results of the PCA, which show that only an explained variance of 14.7% could be calculated for component 2.

**Figure 4:**
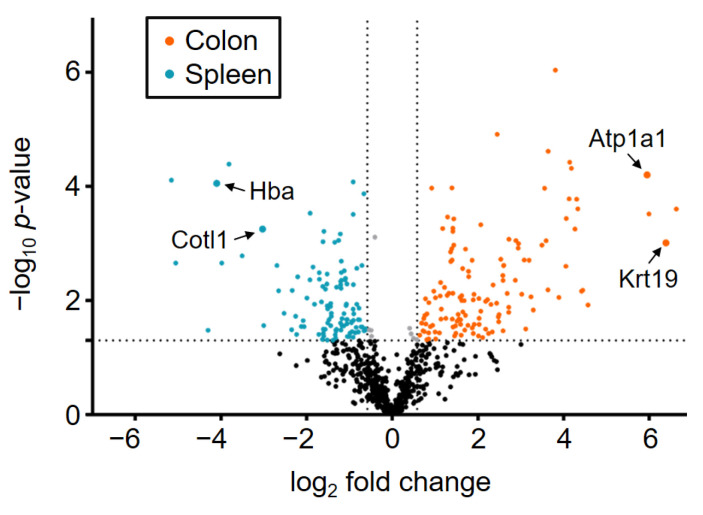
Volcano plot showing the log_2_ fold change values of 715 proteins plotted against their associated −log_10_ *p*-values. Dots representing individual proteins were divided in four different groups: *p*-value > 0.05 (black); *p*-value < 0.05 (grey); *p*-value < 0.05, 1.5-fold higher abundance in colon (orange); *p*-value < 0.05, 1.5-fold higher abundance in spleen (blue). For both sample groups two proteins, being typical higher abundant in colon or spleen tissue, respectively are highlighted and named (see Supplementary Table S4).

*T*-testing results (see Supplementary Table S4) are displayed as a volcano plot in Figure 4 showing the −log_10_ *p*-value against the log_2_ fold change between spleen and colon. The plot is based on 715 proteins, which have only two of three valid values per tissue type. Of these, 466 proteins (shown in black) have no *t*-test significance (*p*-value > 0.05) are shown in black. Based on the size of their change in abundance, *t*-test significant proteins (*p*-value < 0.05) can be divided in three different groups. Wherein 131 significant proteins with 1.5-fold higher abundance in the colon are shown in orange and 110 proteins with 1.5-fold higher abundance in the spleen are colored in blue. Significant proteins with an abundance difference less than 1.5-fold are shown in grey.

**Figure 5:**
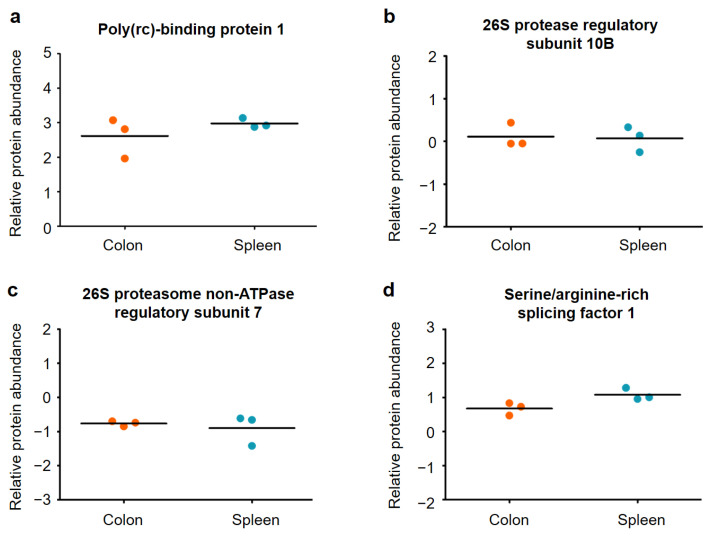
Relative protein abundance plots for (**a**) Poly(rC)-binding protein 1 (Pcbp1), (**b**) 26S protease regulatory subunit 10B (Psmc6), (**c**) 26S proteasome non-ATPase regulatory subunit 7 (Psmd7) and (**d**) Serine/arginine-rich splicing factor 1 (Srsf1) in murine colon and spleen samples. The data in (**a**–**d**) are highlighted in orange for colon tissue and in blue for spleen tissue.

As expected, there are proteins with characteristic abundance in colon and spleen tissue, respectively. These proteins are visible in the volcano plot in Figure 4 as colored dots. Proteins with similar abundances cover about 65% of the colon and spleen tissues. These proteins belong to the basic inventory which every cell requires, independently from its specialization. In the study of Lee et al. (2016) a list of 20 newly identified housekeeping proteins was published showing a uniform protein abundance level (CV < 20%, <1.5-fold change) over 27 different tissue types [29]. In Figure 5 a relative protein abundance plot is shown for four examples of these housekeeping proteins.

**Figure 6:**
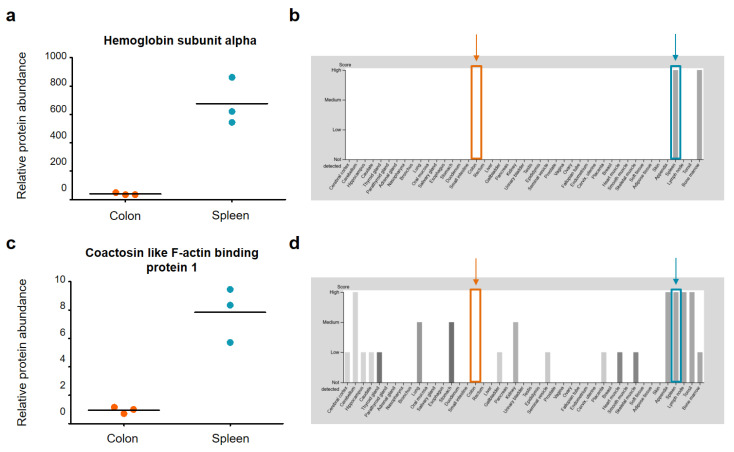
Relative protein abundance plots for (**a**) hemoglobin subunit alpha (Hba) and (**c**) coactosin like F-actin binding protein 1 (Cotl1) in murine colon and spleen samples. The corresponding protein abundance data of the Human Protein Atlas (HPA) [30] is shown in (**b**) for Hba and (**d**) for Cotl1. A total of 44 different tissue types were examined for the protein abundance data of the HPA. The data in (**a**,**c**) is highlighted in orange for colon tissue and in blue for spleen tissue. Images (**b**,**d**) are available from v20.1.proteinatlas.org.

**Figure 7:**
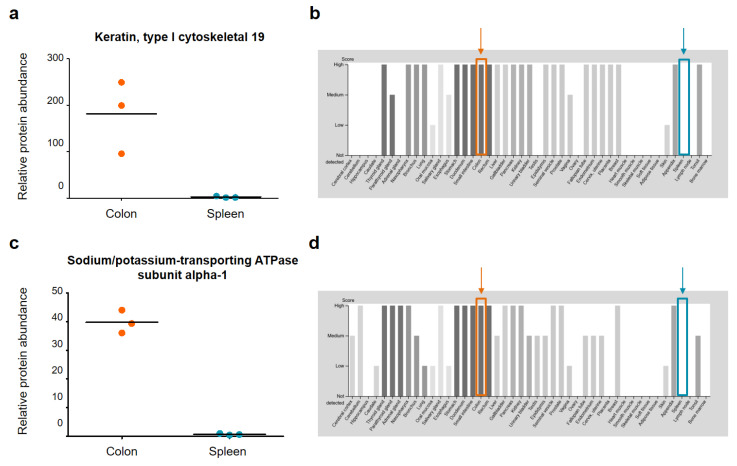
Relative protein abundance plots for (**a**) keratin, type I cytoskeletal 19 (Krt19) and (**c**) sodium/potassium-transporting ATPase subunit alpha-1 (Atp1a1) in murine colon and spleen samples. The corresponding protein abundance data of the Human Protein Atlas (HPA) [30] is shown in (**b**) for Hba and (**d**) for Cotl1. A total of 44 different tissue types were examined for the protein abundance data of the HPA. The data in (**a**–**d**) is highlighted in orange for colon tissue and in blue for spleen tissue. Images (**b**,**d**) are available from v20.1.proteinatlas.org.

Figure 5 shows very similar relative protein abundances for the colon and spleen samples for the four selected housekeeping proteins. Based on this, we can confirm that the specific differences between the colon and spleen samples are indeed due to significantly different protein abundances in the tissues and not due to, for example, different suitability of both organs for tissue sampling via NIRL. For further validation, two example proteins of each sample group with characteristic abundance in the respective tissues have been highlighted and named in Figure 4. For the colon tissue samples, the proteins hemoglobin subunit alpha (Hba) and coactosin like F-actin binding protein 1 (Cotl1) are significantly higher in abundance. The proteins Keratin, type I cytoskeletal 19 (Krt19) and sodium/potassium-transporting ATPase subunit alpha-1 (Atp1a1) show a 1.5-fold higher abundance in spleen tissue compared to the colon tissue samples. For these proteins, the relative protein abundances are compared against the corresponding protein abundance data from the Human Protein Atlas version 20.1 (http://www.proteinatlas.org, accessed on 15 July 2020) [30] in the following Figure 6 and Figure 7.

The relative abundances of both proteins Hba and Cotl1 (see Supplementary Table S4) were plotted for the two different tissues, colon and spleen in Figure 6. Furthermore, the protein abundance data from the Human Protein Atlas for Hba and Cotl1 are shown. Both Hba and Cotl1 show significantly higher protein abundance in spleen than in colon tissue. In the “protein expression overview” of the Human Protein Atlas, the protein abundance data is shown for 44 tissues. Based on the “protein expression overview” for Hba and Cotl1 it is shown that a high abundance of both proteins is characteristic in human spleen tissue. Our results of significantly higher protein abundance of Hba and Cotl1 are corroborated by the human protein abundance data. A high abundance of Hba is characteristic for spleen tissue and bone marrow. In contrast, Cotl1 can be found with high abundances for several tissues such as appendix, lymph nodes and tonsil [30].

Figure 7 shows the relative protein abundance for the proteins Krt19 and Atp1a1 (see Supplementary Table S4) as well as the corresponding protein abundance data from the Human Protein Atlas for 44 tissues. Based on our results with these two example proteins, the relative protein abundance is higher in murine colon tissue than in spleen tissue. The protein abundance data from the Human Protein Atlas show that both Krt19 and Atp1a1 were detected with a high score in colon tissue, whereas no score could be calculated for spleen tissue, which is in accordance with our data. Furthermore, the data from the Human Protein Atlas shows that both proteins are not only highly abundant in colon tissue, but also in many other tissues such as kidney and appendix [30].

## 3. Discussion

In this study, we ablated three samples from fresh-frozen murine colon and spleen at different locations (ablation sites) with a volume of 1.1 × 1.1 × 0.4 mm³ (approximately 0.5 µL). The aerosol plume was condensed and then subjected to differential quantitative mass spectrometric bottom-up proteomics. A total number of 1889 proteins were identified with relative quantitative information, with 1617 proteins in the colon tissue samples and 1207 in the spleen tissue samples (see Supplementary Tables S1 and S2). The results of the differential quantitative proteome analysis display the expected proteomes of colon and spleen tissue and clearly demonstrate the applicability of NIRL for sampling tissues and immediate homogenization for proteomics.

In Table 2 we list ablation parameters and the number of identified proteins of this study together with data of other publications with similar experimental setups, utilizing infrared laser systems with the wavelength of the OH vibration stretching band of water at about 2.94 µm and subsequent LC-MS/MS analysis for mass spectrometric proteomics.

Since the number of identified proteins depends on the parameters of the sampling laser system, tissue type, sample collection approach, sample preparation steps, the applied MS instruments and algorithms for identification of the proteins, a comparison of the number of identified proteins of the different studies does not offer any information about the quality of the different IR-laser based sampling procedures. For instance, if we compared the yield of proteins from muscle tissue and renal tissue analyzed with the same bottom-up proteomics LC-MS/MS method, the total number of identified proteins of muscle tissue would usually be significantly lower, because of the ion suppression effect of high abundant proteins like myosin. For a meaningful comparison, a new study is required, comprising all the different sampling approaches and using the same tissue followed by the same LC-MS/MS based proteomics strategy. Nevertheless, the listed studies show that sampling of different tissues with IR-lasers works well. To enable a higher spatial resolution of sampling of tissue with an IR-laser, the sample volume must be reduced further. With the latest mass spectrometers designed for single cell proteomics, sufficient numbers of identified proteins should be obtainable in the near future. This study, together with that of Wang et al. [21], are steps towards three-dimensional tissue sampling aiming for ultrahigh spatial resolution. Against this background, we want to highlight some aspects of the chronologically listed studies from Table 2, based on the identified proteins.

Compared to our previous studies [4,15,16] the total number of identified proteins are in the same order of magnitude, but with a significantly smaller ablation volume in this study. With regards to the study of Kwiatkowski et al. (2016) [4] the ablation volume was reduced 100-fold down to 0.5 µL. In the work of Kwiatkowski et al. (2016) and Hänel et al. (2018) [15] the aerosol was collected with a cooling trap or a glass fiber filter in combination with a vacuum pump. In this study we used a direct sampling approach in a reflection configuration, similar to studies from Park and Murray [31], who used a microscope slide to directly collect the ablated tissue in a transmission configuration for subsequent matrix-assisted laser desorption/ionization (MALDI) imaging. The increase of the efficiency of aerosol collection by the avoidance of aerosol transport tubing and the improvement of laser parameters of the NIRL setup are the main reasons for this progress. The main drivers for the improvement in the ratio of identified proteins to ablation volume are most likely the high beam quality factor (M² = 2.5), the relatively high applied laser fluence of the 7 ns pulses in combination with the low repetition rate (20 Hz), which minimizes heating.

The high yield of identified proteins in relation to the ablated volume in the study of Pettit et al. (2018) may be achieved because the authors used a mass spectrometer additionally equipped with an ion mobility (IM) mass analyzer. It was also shown, that the applied mass spectrometer yielded up to four times more identified proteins than tandem mass spectrometers without an IM [17].

In summary, it can be stated that high energy picosecond, nanosecond or even microsecond infrared lasers are sufficient for sampling tissues for subsequent bottom-up proteomics. The development of new highly sensitive mass spectrometers is opening the possibility to further decrease the tissue sampling volumes. Since in our new approach we ablate directly from the tissue and collect the sample in a reflection configuration, which enables us to sample in three dimensions (3D) we are not only capable of sampling in three dimensions (3D), but also making sure to always sample fully vaporized material. In contrast to a transmission configuration, there is a risk that material above the ablation area will also be blasted off [20]. Furthermore, we avoid material loss inside the tubing by not using aerosol transport system as described in our previous studies [4,15,16].

In the future, the technique described here may not only be the basis for basic research and investigation of small to even smaller amounts in the sub-nanoliter scale of tissues, but also for diagnostics in the sense of “precision pathology based on proteomics” giving the opportunity to apply high-resolution proteomics for patients [32].

## 4. Materials and Methods

### 4.1. Animals

Mice aged 8–12 weeks old used in this study were on a C57/BL6 background. Mice were kept under specific pathogen free conditions, at an ambient temperature of 20 ± 2 °C, humidity of 55 ± 10% and a dark/light cycle of 12 h.

### 4.2. Ablation Setup

The ablation setup is depicted in Figure 1a and consists of the following devices and optical elements. From the outlet of the pulsed nanosecond infrared laser (NIRL) (Opolette SE 2731, Opotek, LLC, Carlsbad, CA, USA) the beam passes a telescope with two plano-convex lenses (ISP-PX-25-150 and ISP-PX-25-100, ISP Optics Latvia, LTD, Riga, Latvia) for collimation purposes, followed by a 150 mm focusing lens (ISP-PX-25-150, ISP Optics Latvia, LTD, Riga, Latvia) resulting in an elliptical spot with the diameters d_x_ = 165 µm and d_y_ = 135 µm, respectively. The beam propagation ratio (M²) of the NIRL is stated to be about 2.5 by the manufacturer. The spot dimensions were determined using a metal surface as target, colored with a paint stick, and measured with a microscope. A two-inch dual axis scanning mirror (OIM202, Optics in Motion LLC, Long Beach, CA, USA), controlled by a custom-built data acquisition input/output device, was used for transversal (*x*- and *y*-direction) focal displacement. The axial (*z*-direction) focal position can be adjusted by a slight movement of the second telescope lens. The scanning mirror is hit with an offset to the pivot point by the beam allowing the integration of a camera path for distance measurement purposes. The custom-made control software was timed to fit the repetition rate of the NIRL, which was set to the maximum of 20 Hz. Before each sampling procedure, a fresh glass cover slip (H 877, Carl Roth GmbH + Co. KG, Karlsruhe, Germany) was mounted 2–4 mm above the sample, to let the ablated aerosol condense against it during the ablation process.

### 4.3. Laser Parameters and Tissue Sampling

In this study the tunable wavelength of the NIRL was set to 2.9 µm, as a compromise of matching the strong OH vibration stretching band of water at 2.94 µm and to maximize the power output of the wavelength tuning range at 2.9 µm for the ablation process. The pulse energy was measured to be 560 µJ at the sample position, which corresponds to 11.2 mW at the used maximum repetition rate of 20 Hz. The scanning area was set in the custom control software to 1 × 1 mm² and the distance between spots to 200 µm. The ablation time was set to 5 min for all ablations, resulting in 6,000 applied laser shots at each ablation site on the sample.

For this study we sampled three volumes from fresh-frozen murine colon, transversally cut and folded open, as well as fresh frozen murine spleen, with the temperature controlled to −15 °C during the ablation procedure. During ablation, the sampled tissue is transformed into a plume and immediately homogenized before condensing on the glass cover slip (see Figure 1b,c), where a scanning area of about 1 × 1 mm² of the collected condensate is lost due to the scanning of the laser beam.

The condensed homogenate was then collected in three steps, with a pipette filled with 50 µL of 0.1 M triethyl ammonium bicarbonate including 1% sodium deoxycholate (SDC buffer), from the unmounted glass cover slip after each ablation and transferred into a 1.5 mL Eppendorf tube. Afterwards, the samples (dissolved in 150 µL SDC buffer) were stored at −80 °C for further preparation steps.

### 4.4. Determination of the Ablation Volume

A formalin-fixed murine spleen was used as reference for ablation volume measurements to prevent sample deformation during the volume measurement procedure. The extracted volume was determined utilizing a spectral domain optical coherence tomography (OCT) system (OQ Labscope 2.0, Lumedica, Durham, NC, USA) with a central wavelength of 840 nm. The applied OCT imaging volume was 512 × 512 × 512 voxels with each voxel measuring 11.48 × 11.48 × 3.61 µm³ in air. The OCT image data (see Figure 1d) was manually segmented with the open-source segmentation software ITK-Snap 3.8.0 [28] (see Figure 1e). For each ablation, the voxel count and mean volume dimensions (width and depth) were determined (see Figure 2a–e). The values for all three volumes of the reference ablations, including the mean width and depth for each ablation site are listed in Table 1.

### 4.5. Sample Preparation Protocol

The samples were boiled at 99 °C for 5 min to denature proteins. Afterwards, samples were processed with a Probe Sonicator for one cycle at 30% power to destroy DNA and RNA molecules. According to a BCA protein assay (the Pierce^TM^, Thermo Fisher Scientific, Waltham, MA, USA), 5 µg of each sample were diluted to 100 µL with SDC buffer. For reduction of disulfide bonded cysteine residues, 10 mM dithiothreitol (DTT) were added to the samples and they were incubated for 30 min at 60 °C. After that 20 mM iodoacetamide (IAA) was added to the samples for alkylation of the reduced cysteine residues and they were incubated for 30 min at 37 °C in the dark. Trypsin was added at a ratio of 1:100 trypsin to protein for 16 h at 37 °C. To quench the trypsin and precipitate SDC, 100% formic acid was added to a final concentration of 1% formic acid *v*/*v*. The samples were centrifuged for 5 min at 16,000× *g*. The supernatant was collected and dried in a SpeedVac™ vacuum concentrator. The digested samples were stored at −20 °C until further use.

### 4.6. Mass Spectrometric Analysis

The samples were resuspended in 20 µL of 0.1% FA for a final concentration of 0.25 µg/µL. An aliquot of 4 µL was injected into a Dionex Ultimate 3000 UPLC system. Peptides were purified and desalted using an Thermo Scientific^TM^ Acclaim^TM^ PepMap^TM^ 100 C18 Nano Trap pre-column (100 µm × 2 cm, 100 Å pore size, 5 µm particle size) and transferred to a Thermo Scientific^TM^ Acclaim^TM^ PepMap^TM^ 100 C18 column (75 µm × 50 cm, 100 Å pore size, 2 µm particle size) for chromatographic separation. Peptide elution was achieved by applying an 80-minute run for each sample with a flow rate of 0.3 µL/min at 45 °C using a gradient with Eluent A consisting of 0.1% FA and eluent B of 0.1% FA in 90% acetonitrile starting with 2% solvent B. Solvent B was increased to 30% in 65 min followed by a linear gradient elevating the concentration to 90% in 70 min. Finally, the concentration of buffer B was reduced to 2% after 70.1 min. The eluting peptides were analyzed on a Quadrupole Orbitrap hybrid mass spectrometer (QExactive, Thermo Fisher Scientific, Waltham, MA, USA). Here, the ions that were responsible for the 15 highest signal intensities per precursor scan (1 × 10^6^ ions, 70,000 resolution, 240 ms fill time) were analyzed by MS/MS (HCD at 25 normalized collision energy, 1 × 10^5^ ions, 17,500 resolution, 50 ms fill time) in a range of 400–1200 *m*/*z*. A dynamic precursor exclusion time of 20 s was used.

### 4.7. MS Data Processing

Obtained raw data from the LC-MS/MS measurement of peptides were processed with MaxQuant (version 1.6.2.10). For protein identification, a reviewed murine Swissprot FASTA database without isoforms (status August 2020) containing 17,053 proteins was generated. The measured MS2 spectra were searched with the Andromeda algorithm against the theoretical fragment spectra of tryptic peptides from the data base. The carbamidomethylation of cysteine residues was set as a fixed modification. Oxidation of methionine, protein N-terminal acetylation and the cyclisation of N-terminal glutamine to pyroglutamate were set as variable modifications. Peptides with a minimum length of 6 amino acids and a maximum mass of 6000 Da were accepted with a mass tolerance of 10 ppm. Only peptides with a maximum of two missed trypsin cleavage sites were considered. The error tolerance was set to 20 ppm for the first precursor search and to 4.5 ppm for the following main search. Fragment spectra were matched with a 20 ppm error tolerance. Using a reverted decoy, the peptide database approach was set for protein identification to a false discovery rate (FDR) value threshold of <0.01. Label free quantification (LFQ) was performed with an LFQ minimum ratio count of 1. For quantification, all identified razor and unique peptides were considered. The label minimum ratio count was set to 1. Only unmodified peptides were used for quantification.

### 4.8. Statistical Data Analysis

Statistical analysis was performed using Perseus software (version 1.5.8.5). The quantitative data of 1889 proteins (see Supplementary Table S1) were transformed into log_2_ values and further median normalized per sample (see Supplementary Table S2). Principle component analysis (PCA) was performed for an overall sample amount of 6 samples including three murine colon and spleen samples. Therefore, protein data were filtered for no missing values resulting in 501 proteins (see Supplementary Table S3). Statistical testing between murine spleen and colon samples was performed using a two sample *t*-test based on 715 proteins, which were found in two of three samples per tissue type (see Supplementary Table S4). Only proteins identified with a *p*-value < 0.05 and a 1.5-fold change were considered as statistically significantly different in abundance among the compared groups. Resulting proteins of the two sample *t*-test (*p*-value < 0.05, 1.5-fold change) between colon and spleen were further analyzed using the Human Protein Atlas (status July 2021).

## 5. Conclusions

In our study we demonstrated the ablation of both murine colon and spleen tissue reproducibly with the NIRL. An ablation volume of 1.1 × 1.1 × 0.4 mm³ was sampled, determined with OCT measurements. Based on the results of differential quantitative proteomics, colon and spleen samples could be clearly distinguished. A comparison of the relative abundances of two example proteins per tissue type was carried out. These proteins show significant abundance differences between both tissues and are in accordance with the corresponding Human Protein Atlas data. The yield of proteins is sufficient to identify proteins with significantly different protein abundance, which makes it suitable for biomarker identification approaches based on mass spectrometric proteomics. In conclusion, we demonstrate that a wavelength tunable NIRL is suitable for sampling prior to the differential proteomic analysis of tissues. Additionally, we show that the developed laser setup and aerosol collection method enables a miniaturized analysis, pointing towards spatially resolved proteomic analysis.

## Figures and Tables

**Table 1:** Ablation volumes and dimensions of the three ablation sites of a formalin-fixed murine spleen, determined with optical coherence tomography (OCT) for reference. Volume was determined based on the voxel count and the dimensions (mean width and depth) based on random distance measurements of the segmentation.

Ablation Site ${}^{†}$	Ablation Volume	Mean Width	Mean Depth
[Label Color]	[µL]	[µm]	[µm]
Red	0.38	1,148	397
Green	0.53	1,205	401
Blue	0.40	1,091	404

${}^{†}$ Labels are shown in Figure 1e.

**Table 2:** Parameters and identified proteins of different studies sampling different tissues with an IR-laser with 2.94 µm wavelength and analyzing the samples with mass spectrometric bottom-up proteomics.

Author	Pulse	Laser	Ablation	Ablation	Ablation	Tissue Type	Identified
	Width	Fluence	Area	Depth	Volume		Proteins
	Regime	[J/cm²]	[mm²]	[mm]	[µL]		
Hahn et al. (this study)	ns	6.34	1.1 × 1.1	0.4	0.5	Colon (mouse)	1617
						Spleen (mouse)	1207
Pulukkody et al. 2021 [20]	ns	4.6 ± 2.1	2 × 2	unknown	unknown	Biofilm (plankton)	400
Wang et al. 2020 [21]	ns	2	7	0.01	0.07	Brain (rat)	720
			4		0.04	Midbrain (rat)	439
Krutilin et al. 2019 [16]	ms	40–60	4 × 4	unknown	unknown	Liver (rat)	581
						Muscle (rat)	883
	ps	1	4 × 4	unknown	unknown	Liver (rat)	926
						Muscle (rat)	642
Pettit et al. 2018 [17]	ns	2.7	2 × 2	0.05	0.2	Brain Q1 ${}^{†}$ (rat)	1674/393 ${}^{‡}$
						Brain Q2 ${}^{†}$ (rat)	1723/379 ${}^{‡}$
						Brain Q3 ${}^{†}$ (rat)	1322/326 ${}^{‡}$
						Brain Q4 ${}^{†}$ (rat)	1575/365 ${}^{‡}$
Hänel et al. 2018 [15]	ms	37.00	3 × 3	unknown	unknown	Tumor (mouse)	1922
						Liver (mouse)	1811
						Ovary (mouse)	2099
Kwiatkowski et al. 2016 [4]	ps	3.39	5 × 5	2	50	Pancreas (rat)	1743
						Tonsils (human)	2085
Donnarumma and Murray	ns	3.00	1.2 × 1.1	0.05	0.066	Cerebellum (rat)	250
2016 [14]						Midbrain (rat)	95

${}^{†}$ Q1–Q4 refer to different quadrants in the brain slice (thickness of 50 µm); ${}^{‡}$ Maximum of three ablated sections with/without ion mobility mass analyzer.

## Data Availability

Mass spectrometric data generated in this study can be accessed through the ProteomeXchange Consortium via the PRIDE partner repository with the dataset identifier PXD027700.

## Supplementary Materials

The following are available online at https://www.mdpi.com/article/10.3390/ijms221910833/s1.
